# The Responses of Aquatic Plants to Water-Level Fluctuation: A Review

**DOI:** 10.3390/plants15172673

**Published:** 2026-08-31

**Authors:** Ai-Ping Wu, An-Guo Gao, Hui-Wen Li, Jing-Rui Yuan, Gui-Xiang Yuan, Hui Fu, Jie Zhang, Yong Zhang, Song-He Zhang, Yan-Hong Wang, You-Zhi Li, Zhen-Rong Huang

**Affiliations:** 1 Ecology Department, College of Environment and Ecology, Hunan Provincial Key Laboratory of Rural Ecosystem Health in Dongting Lake Area, Hunan Agricultural University, Changsha 410128, China; gaoanguo96@stu.hunau.edu.cn (A.-G.G.); lhw@stu.hunau.edu.cn (H.-W.L.); yuanjr0815@hunau.edu.cn (J.-R.Y.); yuangx987@163.com (G.-X.Y.); fuhui@hunau.edu.cn (H.F.); youzhili2020@hunau.edu.cn (Y.-Z.L.); 2 Hunan Institute of Water Resources and Hydropower Research, Changsha 410125, China; zj15084854067@163.com (J.Z.); z18569032878@163.com (Y.Z.); 3 Key Laboratory of Integrated Regulation and Resource Development on Shallow Lakes, Ministry of Education, College of Environmental Science and Engineering, Hohai University, Nanjing 210098, China; shzhang@hhu.edu.cn; 4 School of Forestry and Bio-Technology, Zhejiang Agriculture and Forestry University, Hangzhou 311300, China; wangyanhong@zafu.edu.cn

**Keywords:** macrophyte, aquatic plants, water-level fluctuation, survival strategy, response, adaptation, water-level regulation, flooding, drawdown

## Abstract

Water-level fluctuation (WLF) is a key factor disturbing aquatic ecosystems, particularly aquatic plants (macrophytes in this review). These organisms are exceptionally sensitive to WLF as it profoundly governs their spatial distribution, productivity, species richness, community composition and successional trajectories. In this review, we synthesized recent advances regarding the impacts of WLF on the growth environment, survival strategies, and the integrated morphological, physiological, and reproductive responses of aquatic macrophytes. While existing research has predominantly focused on flooding-related WLF responses and freshwater macrophytes, the ecological consequences of drawdown and the responses of marine macrophytes remain comparatively underexplored. Despite the adverse alterations in sediment dynamics, light availability, hydrostatic pressure, pollutant concentrations, wind and wave exposure, dissolved oxygen levels and nutrient concentration conditions under WLF, aquatic macrophytes can demonstrate remarkable adaptive plasticity. This resilience was mediated through rapid adjustments in survival strategies, coupled with morphological traits, physiological processes and reproductive modifications. While their adaptive capacities were limited, and varied depending on life-forms, species or the WLF amplitudes, fluctuations in water level often precipitated rapid shifts in dominant species or even community succession over short timeframes. Consequently, while moderate WLF might confer ecological benefits, prolonged or extreme WLF posed substantial threats to aquatic vegetation. Synthesizing these findings, we developed a conceptual model diagram integrating the multifaceted responses of aquatic macrophytes to flooding-induced WLF. Future research should prioritize investigating the ecological consequences of drawdown-induced WLF and the responses of marine macrophytes—areas that remain comparatively underexplored. Furthermore, implementing refined water-level-management regimes could help optimize the good functions and services delivered by aquatic ecosystems.

## 1. Introduction

### 1.1. Background

Water-level fluctuation (WLF) is a widespread and intense (in some regions) natural disturbance in aquatic ecosystems [1,2,3], although the fact is that the ecological integrity of such ecosystems relies on natural WLF patterns [4]. This is due to the substantial temporal variability in snowmelt, rainfall and evapotranspiration across scales ranging from hours to years [5,6], which is natural WLF caused by natural events. The impact of WLF disturbance intensifies under increasing climatic stochasticity, particularly given the rising intensity and frequency of exceptionally extreme rainfall and drought events driven by climate change [2,7,8,9]. Furthermore, these aquatic ecosystems face substantial anthropogenic pressures. Hydrological modifications, primarily involving the construction of canals, levees, weirs, plugs and dams for flow regulation and hydropower generation [10,11,12,13], create regulated WLF regimes that are themselves a direct consequence of human activities. For example, over 3700 large (more than 1 MW) and 82,800 smaller (less than 1 MW) hydropower plants are either operational or under construction throughout the world [14,15]. This expansion is particularly pronounced in developing countries, which harbor substantial untapped hydropower potential. This trend is largely driven by the dual imperatives of affordable, low-carbon, clean energy and the escalating demands of economic growth and population pressure [13,16]. In most instances, the intensity and frequency of anthropogenically driven WLF exceed those caused by natural variability in aquatic ecosystems, thereby posing significant ecological risks [17,18]. Consequently, aquatic ecosystems typically face concurrent pressures from both natural and human-induced WLF [6,19,20], making their impacts a focal point of research in recent decades [21,22,23,24,25]. However, Evtimova and Donohue [26] argue that our understanding of WLF’s ecological effects remains considerably limited compared to other anthropogenic stressors, although WLF represents a pervasive and dominant pressure on global aquatic ecosystems [27].

Usually, the structure, function, goods and services of aquatic ecosystems are strongly influenced by WLF, and the health and integrity of these ecosystems are evidently damaged by any significant WLF [28,29,30,31]. Previous researchers suggest that intense WLF conditions typically drive reductions in species diversity, homogenize community composition, and curtail both the extent and productivity of macrophyte vegetation, ultimately diminishing overall aquatic biodiversity [32,33,34,35,36]. Prolonged or intensified flooding can, ultimately, cause irreversible damage to submerged vegetation, often culminating in plant mortality. Aquatic plants (macrophytes in this review), as the main primary producers in aquatic ecosystems, function well and are important in maintaining ecosystem health, stability, and trophic integrity [37,38]. These organisms, however, remain exceptionally sensitive to WLF [39,40], although they have evolved a suite of specialized and specific plastic traits, morphology and physiology to adapt to aquatic environments [41,42,43,44,45]. Therefore, the WLF effects on aquatic macrophytes have emerged as a hot topic in aquatic ecology, resulting in a huge quantity of articles [6,20,46,47,48]. This research domain has exhibited remarkable growth in recent decades, yet comprehensive and up-to-date systematic reviews remain surprisingly scarce, despite the burgeoning literature [40].

### 1.2. Gap in Current Reviews

Blom and Voesenek [47] systematically examine the survival strategies of plants in flooding zones, analyzing responses to inundation stress across multiple levels. Complementing this, Leira and Cantonati [39] mainly focus on the temporal and spatial trends of WLF and their primary effects on macrophytes, demonstrating that WLF effects on both aquatic macrophytes and ecosystems are very complex. Building on empirical evidence, Webb [40] constructed the causal relationships between water regime components and their effects on plant characteristics. Their results indicate that the deleterious negative effects on plant traits increase with the increasing magnitude of the hydrological perturbation. Extending this focus to the littoral zone, Carmignani and Roy [18] analyze the effects of winter water-level drawdowns on abiotic environments and biotic organisms. The results show that the species richness of macrophyte and benthic invertebrates is greatly reduced by the annual winter water-level drawdowns, with r-selected species being disproportionately favored. Scaling up, Rolls [49] determine the spatial scale biodiversity responses of freshwater species to natural hydrological regimes and construct a conceptual model to analyze these effects. Complementing this focus on natural flows, Bejarano [50] conducted a comprehensive literature review on the negative effects of hydropeaking on riverine plants, including aquatic macrophytes. Crucially, this work advocates for maintaining hydropeaking operations below critical thresholds to safeguard riparian plant communities. Recently, Jiang and Chui [51] synthesized the biological responses of constructed wetlands to changing hydrological conditions. However, a major knowledge gap remains in terms of summarizing (1) how WLF affect the environmental conditions of aquatic macrophytes, and (2) how aquatic macrophytes adapt to WLF through integrated survival strategies encompassing morphological, physiological and propagative responses.

### 1.3. Methods

We constructed our database via a comprehensive literature search in the Web of Science (Clarivate Analytics, Ann Arbor, MI, USA) in June 2024, using the search string: “Title (TI) = (water depth OR water table OR water level OR Hydrolog*) AND Topic (TS) = (Aquatic plant OR Aquatic species OR Macrophyte OR Hydrophyte)”. This initial search yielded 7523 English-language articles after removing duplicates. Titles and abstracts were screened, followed by full-text reviews, to assess compliance with our inclusion criteria. Studies were included if they (i) investigated aquatic plant responses to water-level fluctuations and (ii) focused on macrophyte species. Consequently, 191 studies from the database, plus an additional 14 articles identified through other sources, met the criteria (see Supporting Material). These studies were categorized based on plant survival strategies, as well as integrated morphological, physiological, and reproductive responses. Finally, we synthesized and summarized these four distinct response types.

### 1.4. Objectives

It is well established that WLF cannot affect aquatic macrophytes alone but in combination with other factors, such as sediment type, light, wind and wave action, and flow velocity [20,52,53,54]. The ecological impacts of WLF on aquatic macrophytes are further mediated by key hydrological attributes: amplitude, frequency, duration, rate of change, and temporal timing [39,47,55,56]. Through a systematic review of the literature spanning recent decades, we examined the multifaceted relationships between WLF and diverse aquatic macrophytes (including emergent, rooted floating-leaved, and submerged but not un-rooted floating-leaved (also free-floating) plants). The objectives of the study were to (1) determine how WLF modify the environment governing aquatic macrophyte establishment and growth, (2) characterize macrophyte responses across survival strategies, morphological adaptation, physiological adjustments, and propagative mechanisms, and (3) develop an integrative conceptual model illustrating plant responses to elevated water levels.

## 2. WLF Effects on Growth Environment of Aquatic Macrophytes

**Figure 1:**
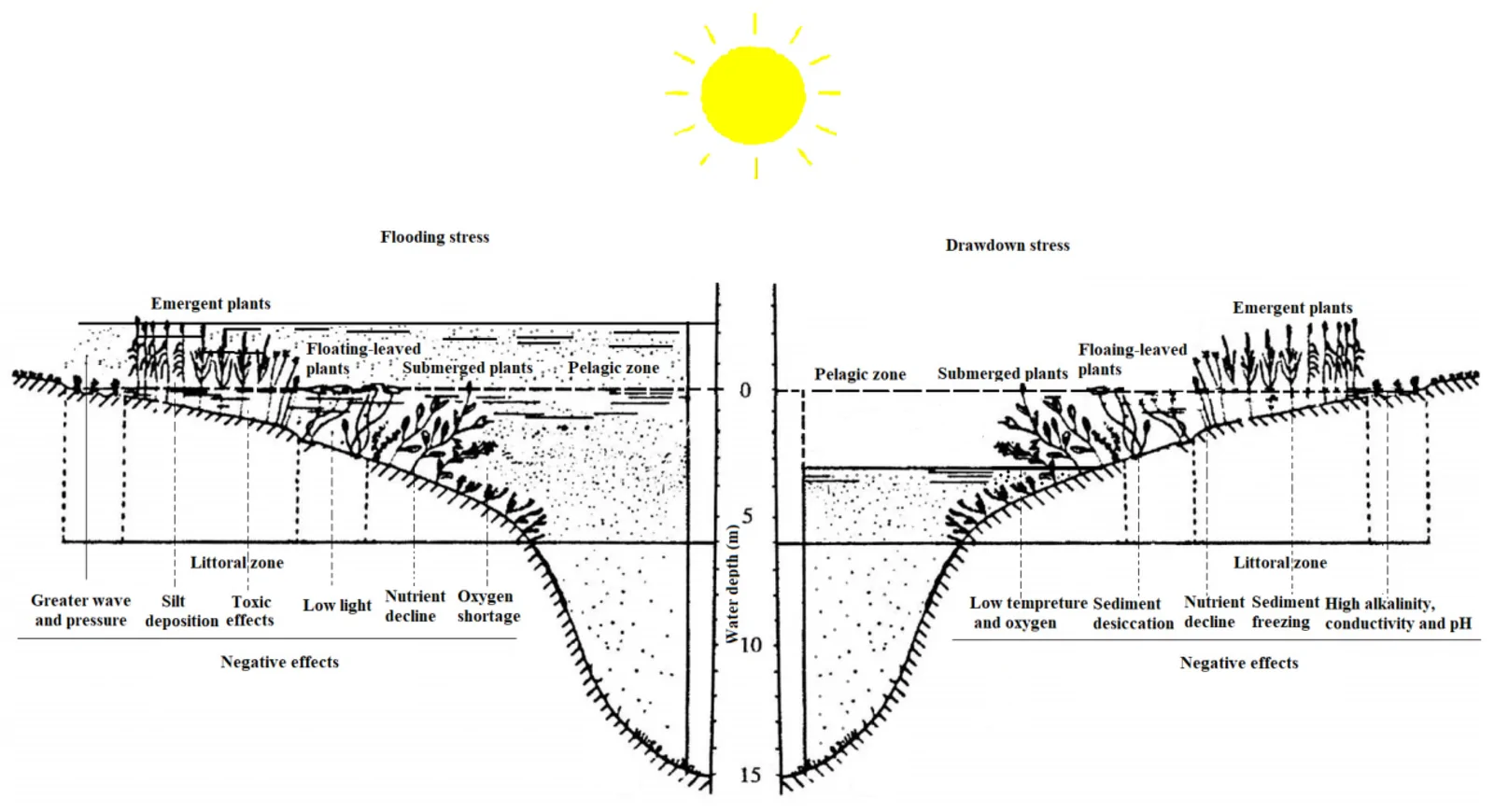
The negative effects on abiotic environments where macrophytes grow under water flooding or drawdown conditions.

Water depth, a direct manifestation of WLF, serves as an integrative character reflecting the combined influence of multiple environmental factors, including illumination intensity, water pressure, pollutant loads, and the availability of oxygen and nutrients. Accordingly, numerous studies suggest that WLF can cause the alternation of biodegradation [57], oxygen concentrations [58,59], gas exchanges on the soil-water interface [60], and groundwater hydrodynamics [61]. Furthermore, WLF facilitates the redistribution of various contaminants across liquid, gaseous and solid phases [62]. Crucially, these fluctuations significantly govern the migration, transformation, toxicity and degradation of a spectrum of biogeochemically relevant substances. As illustrated in Figure 1, these include carbon [63], nitrogen [64], and phosphorus [18,47,65], as well as specific pollutants such as light non-aqueous phase liquids [66], benzene series [67,68], total petroleum hydrocarbons [69] and so on.

### 2.1. Environmental Impacts: Drawdown

#### 2.1.1. Sediment Indices

Water drawdowns typically induce the erosion of surface sediments in the upper littoral zone, causing sediment coarsening, while sediments in deeper zones often become finely pulverized [70,71,72,73]. Furthermore, drawdown-exposed sediments are particularly susceptible to desiccation, freezing and snow accumulation [74]. Therefore, submerged sediments in regulated (drawdown) aquatic ecosystems are more disturbed by snow and ice than those in unregulated ones [18,75,76,77,78]. On the contrary, an insulating snowpack can prevent sediments from freezing and scouring [18,79]. The nutrient storage capacity of sediments declines because of sediment coarsening and increased bulk density processes [80], subsequently restricting sediment-water nutrient flux [78] during drawdown periods. Conversely, the nutrient release of these exposed sediments may be enhanced by regulated drawdowns after rewetting [81].

#### 2.1.2. Other Indices

Moreover, some chemical structures of sediments may be changed with water drawdown [18]. At first, exposed sediments exhibit significantly higher phosphate adsorption capacity compared to submerged sediments, though this capacity decreases and may ultimately disappear with prolonged oxidation and desiccation [82]. Despite these insights, empirical studies examining drawdown-induced variations in sediment ion or element content are very limited [18,83]. Similar to nutrient release, calcium, silicon and potassium content in sediments also increases significantly upon rewetting, resulting in the increase in alkalinity, conductivity and pH values in water [83,84]. Meanwhile, water-column dissolved oxygen concentration declines in winter with drawdowns due to water volume loss [85,86,87]. The thermal effects of winter drawdowns, however, are contingent upon the local climate and the morphometry of aquatic ecosystems. For example, littoral zones often exhibit lower temperatures during winter drawdown periods compared to normal water zones [18]. Ecologically, aquatic ecosystems may change from clear to turbid because the vegetations are greatly destroyed or even lost under water drawdowns [88]. Conversely, extreme low water in a turbid aquatic ecosystem can kill fish because of anoxic conditions due to fish accumulating or water shortage, which results in clear water conditions and subsequent dominant vegetation after a heavy rain [89].

### 2.2. Environmental Impacts: Flooding

#### 2.2.1. Oxygen and Nutrient Shortage

Upon flooding, water permeates exposed sediment soil pores, and then gas diffusion is blocked severely, resulting in a lower oxygen supply around plant roots and shoots and light deprivation [20,47,65]. Concurrently, the soil redox potential declines sharply since plants and soil organisms consume the remaining oxygen gradually as flooding continues [6,47]. Simultaneously, the nutrient elements available to plants strongly reduce because some important microbial processes, such as nitrification, are interrupted [47,65,90]. Consequently, uptake of nitrogen and other vital nutrients is significantly reduced, manifesting in both lower total amounts of nutrient and tissue nutrient contents within the plants [90,91]. Paradoxically, higher phosphate and ammonium concentrations in pore water are observed at higher water levels [92,93], which is potentially attributable to a substantial release of nutrients originating from chemical changes and enzyme activation (e.g., P-mineralizing enzyme) during repeated drying and rewetting cycles [94]. In contrast, some dissolved nutrient concentrations, particularly ammonia and nitrate, decline when water levels increase because of a dilution effect [95].

#### 2.2.2. Toxic Effects

Under flooding conditions, plants inevitably experience severe anoxia once the remaining oxygen is depleted [6,65]. This redox disequilibrium greatly inhibits many chemical transformation processes, most notably the reduction in ferric and manganese oxides and the transformation from nitrate to nitrogen or ammonium ions [47,65]. However, reduced ions and toxic byproducts generated through anaerobic respiration by plants and other organisms accumulate progressively, creating phytotoxic conditions [40,47]. Crucially, this anaerobic condition severely suppresses root formation, respiration, and leaf initiation and expansion, while simultaneously accelerating root decay, and leaf senescence and abscission [91,96,97]. These antagonistic processes markedly impair both shoot and root biomass accumulation, ultimately compromising plant viability [91,96,97].

#### 2.2.3. Other Negative Effects

Under high water-level conditions, organic matter and fine particles, such as clay and silt, are effectively removed by water circulation [98]. As floods subside, however, heavy silt deposits often accumulate, potentially burying plants either partially or completely. This burial stress reduces plant survival and decreases plant growth [99,100,101,102]. Furthermore, elevated water levels amplify wave-induced physical disturbance [103,104]. Concurrently, increased water depth significantly attenuates light, substantially reducing photosynthetically active radiation, which precipitates abrupt declines in photosynthetic activity and heightened plant mortality [105,106]. This, in turn, often triggers a regime shift toward a turbid water state in the aquatic ecosystem, primarily driven by a great loss of macrophytes, particularly submerged species [107]. Paradoxically, despite the simultaneously deficiencies in light, oxygen, and nutrients, along with elevated hydrostatic pressure and accumulations of cover materials and toxic substances, certain studies report relative stability in terms of water temperature, salinity, nutrient levels and disturbance regimes under deeper water conditions [108].

## 3. Plant Responses to WLF

**Figure 2:**
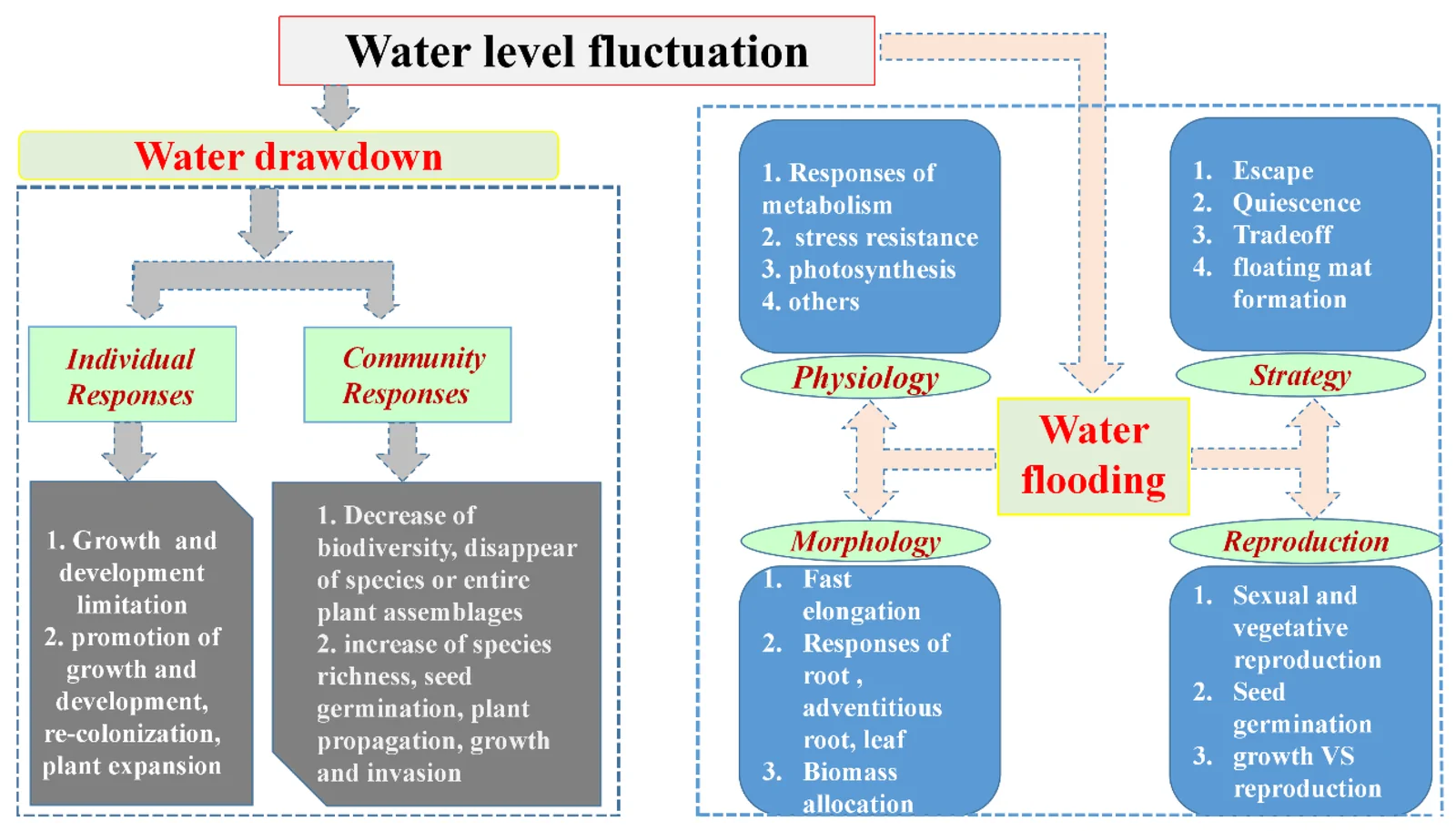
A framework of the macrophyte responses to water flooding or drawdown conditions.

In this section, we sorted macrophyte responses to WLF into two distinct paradigms: responses to drawdown and responses to flooding. We described them in detail in the following two sections, which are followed by a comprehensive synthesis and visual representation of the defined responses in Figure 2.

### 3.1. Plant Responses: Drawdown

#### 3.1.1. Individual Effects

Overall, the coarse, nutrient-poor sediments characteristic of exposed drawdown zones render these areas generally unsuitable for macrophyte colonization or growth, particularly along steep littoral gradients [109]. Consequently, macrophytes establishment is largely restricted to permanently submerged zones, where sediments prevail that are organically enriched and which have sufficient nutrients [109]. However, plant responses to water drawdown are highly heterogeneous, and are mediated by differences in morphological plasticity, life-history strategies, stress tolerance, etc. [18]. Compared to normal zones, plant density, biomass, production and coverage exhibit significant declines in drawdown-exposure zones [71,84,110,111,112,113]. Nevertheless, reducing the amplitude of winter drawdown can facilitate macrophyte biomass accrual in novel wetted zones [18,114]. Importantly, empirical evidence suggests that while the biomass of non-rooted aquatic species may decline, drawdown regimes often promote the productivity and diversity of rooted plants [18,114].

Macrophytes can re-colonize drawdown-exposure zones when growing conditions are suitable for the growth and settlement of new seedlings or residing individuals [115,116]. Generally, emergent species are eliminated when water levels are high, yet many exhibit regenerative capacity upon drawdown. This recovery is facilitated by the new exposure of substrates, which create favorable conditions for emergent plant expansion [39]. For instance, Crisman et al. document a particularly striking example of this phenomenon in Lake Koronia, Greece, where *Phragmites australis* expanded its dominant area more than eight times from 1977 to 2005 in response to sustained water-level drawdown [117]. However, the long-term persistence of these populations in such exposure zones is closely related to the intensity and frequency of drawdown events, as well as the plant resilience to associated stressors, such as the above-mentioned desiccation, freezing and sediment erosion [18,118].

#### 3.1.2. Community Effects

Additionally, the aquatic species assemblages and community structures are also altered by an annual winter drawdown [119,120]. For example, aquatic macrophytes diversity is decreased sharply if the drawdown amplitudes are large enough [109,119] and some species will disappear or the entire macrophyte assemblages be lost under extreme drawdown amplitudes [121,122]. By contrast, moderate drawdown amplitudes typically foster enhanced plant richness [119,123,124], with increased macrophyte species richness in response to drawdown occurrences in some places [125]. This facilitative effect arises because drawdowns act as a prerequisite for successful seed germination and seedling survival among aquatic taxa, particularly emergent species [123,126]. Moreover, drawdown-induced disturbances can alleviate competitive dominance by superior competitors, thereby releasing niche space and promoting coexistence among a greater number of plant species [127].

Under drawdown conditions, species diversity of submerged macrophytes typically increases, whereas that of emergent and floating-leaved plants shows no comparable response [123]. This divergence is partly because of enhanced light availability for submerged species. Accordingly, regular annual drawdowns foster the development of distinct, drawdown-tolerant plant assemblages [18,128]. Previous research suggests that plant species with ruderal or semi-ruderal life-history strategies, characterized of polymorphic growth forms, amphiphytic adaptations, and free-floating characteristics, are particularly favored in drawdown-affected aquatic ecosystems [84,121,124]. This selectivity arises because favorable springtime conditions promote the rapid growth and establishment of ruderal species, while concurrently constraining species with alternative strategies [71]. Usually, sediment desiccation caused by drawdown may facilitate seed germination [98] and subsequent plant propagation or growth [129] during the following spring growing season. Notably, brief drawdown periods can also improve vegetative fragment survivorship and the growth of invasive species such as *Elodea canadensis* and *Elodea nuttallii* [130].

### 3.2. Plant Responses: Flooding

#### 3.2.1. General Responses

Under submergence conditions, flooded aquatic macrophytes (both individual plants and plant communities) mainly suffer from stressors including intensified wave action, reduced light availability, toxicant accumulation, and hypoxic conditions [56,131,132,133,134]. To survive these flooding conditions, aquatic macrophytes not only develop their specific survival strategies but also adapt to flooding with their own physiological, morphological and reproductive adaptations [135,136]. Adaptive responses are distinctly different among different species or life-forms [55,137,138,139]. Crucially, the capacity for adaptation is strictly constrained by the amplitude and duration of WLF; indeed, normal growth, development, propagation and survival of aquatic macrophytes are viable only within a narrow WLF window [140,141,142]. Therefore, we describe these four adaptive responses (survival strategy, morphological, physiological and reproductive responses) separately in the following sections.

#### 3.2.2. Survival Strategy

**Figure 3:**
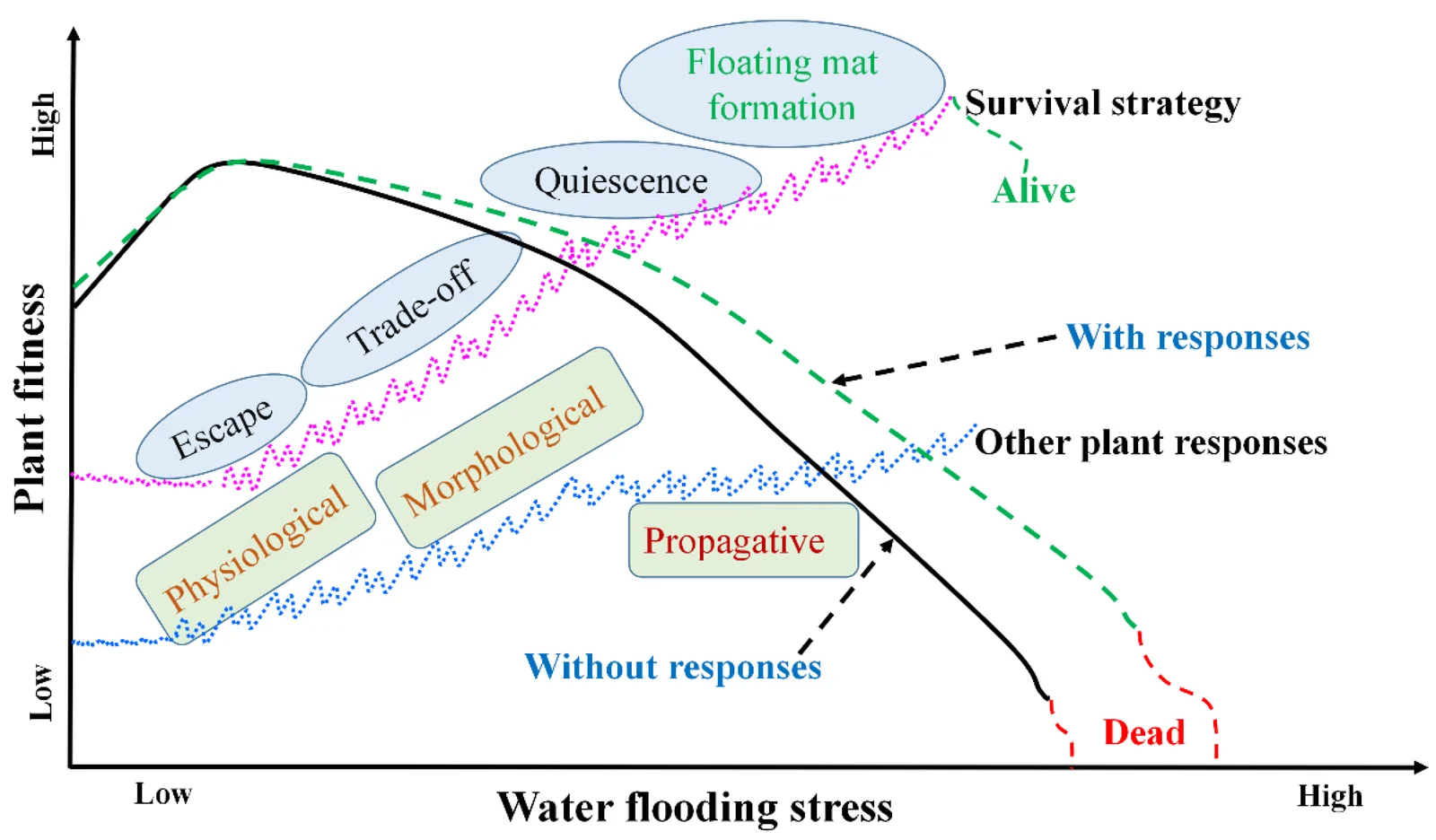
A model of macrophyte fitness under water flooding stress with or without plant responses.

Based on our literature synthesis, we identified four principal survival strategies employed by aquatic macrophytes to withstand flooding, and these are categorized by flooding amplitudes [20,47]. To illustrate these adaptive mechanisms, we constructed an integrative conceptual model (Figure 3). Aquatic macrophytes may not have to respond to an intangible flooding, which is dependent on different species or life-form types, and macrophytes may be stimulated to grow to achieve a better fitness status [143,144,145]. With the increase in flooding amplitude where submerged species suffer from a little or slight flooding stress or emergent and rooted floating-leaved species are partly submerged, plants typically employ an *escape strategy* characterized by accelerated shoot elongation. This involves rapid extension of stems, leaves, branches, or other aerial structures to maintain access to atmospheric oxygen [146,147]. This active escape strategy allows plants to tolerate certain endurable stressors while exhibiting specific morphological adjustments: increased plant height (younger shoots), enhanced branch length or shoot internode elongation, longer petioles and expanded laminae. These growth responses can manifest even during brief flooding events [148], although some plants may concurrently reduce their branch numbers [149,150,151]. The underlying mechanism driving this escape response is the stimulation of cell growth or cell division [152,153].

As flooding amplitudes increase further, subjecting submerged species to moderate flooding stress or completely submerging emergent species and rooted floating-leaved species, aquatic macrophytes typically adopt a *quiescence strategy*. This involves remaining metabolically inactive underwater until water levels recede [65,154,155]. Unlike the active escape strategy, this passive survival approach entails prolonged exposure to intense flooding stress. Through this quiescence response, aquatic macrophytes can maintain minimal metabolic function using stored energy reserves to endure extended submergence, a situation that is functionally analogous to hibernation in terrestrial animals [155,156]. Relatively few aquatic species are reported to adopt this *quiescence strategy*; documented examples include *Rumex crispus* [157], *Oryza sativa* [158], *Ranunculus repens* [159], *Eleocharis cellulosa* [160] and *Acorus calamus* [137]. By contrast, Manzur [136] report that *Lotus tenuis* can choose an alternative adaptive strategy, dynamically switching between *escape* and *quiescence strategies* based on flooding stress intensity—an adaptive flexibility we term the *tradeoff strategy*. To date, this dynamic strategy selection mechanism remains sparsely documented in the literature.

Recent observations indicate that *Zizania latifolia* can dominate the emergent plant community in Lake Erhai following sustained, extreme water-level rises, i.e., those exceeding previously recorded fluctuations [20]. This ecological success is attributed to the species’ exceptional capacity for floating mat formation, a phenomenon we define herein as the *floating mat formation strategy*, correspondingly [20]. While this strategy represents a relatively passive survival mechanism, it enables plants to mitigate extreme water-level stress while remaining exposed to concurrent abiotic stressors such as wind and wave action [20,48]. Importantly, the *floating mat formation strategy* confers significant survival advantages, even during extreme flooding events. Many other emergent genera, such as *Carex*, *Cladium*, *Cyperus*, *Phragmites*, *Scirpus*, *Typha*, *Vossia* and *Zizania* [20,161], demonstrate similar floating mat development capabilities. However, the relationship between their floating mat formation capacity and survival strategies under flooding stress remains insufficiently understood and warrants further investigation. We emphasize that aquatic macrophytes typically employ multiple survival strategies during flooding, although one dominant survival strategy (such as floating mat formation) may overshadow the expression of other adaptive mechanisms.

#### 3.2.3. Morphological Responses

##### Fast Elongation

Macrophyte morphological responses to WLF primarily manifest as structural and anatomical plasticity across root (including adventitious roots), stem, shoot, and leaf systems, as well as biomass reallocation between roots and shoots or among organs [40,48,138,139]. This morphological plasticity is generally considered to correlate inversely with plant lignin content, with lower lignin concentrations facilitating greater morphological adaptability [153,162]. Upon the onset of flooding stress, aquatic macrophytes have to strategically optimize resources between shoot and root growth. Woody plants typically prioritize leaf production over wood tissue growth [163], while herbaceous species maintain leaf growth but reducing investment in root and stolon development [164,165].

Under flooding conditions, the rapid elongation of aquatic macrophytes, manifested as an increase in plant height through accelerated stem and leaf growth, is a well-documented phenomenon. This vertical growth response enables aquatic macrophytes to reach the water surface, thereby mitigating hypoxic stress and optimizing photosynthetic activity and survival [18,40,166]. This elongation response is particularly stimulated by low light conditions characteristic of deep water, mimicking the shade-avoidance syndrome observed in terrestrial plants [167,168]. However, significant declines in shoot biomass, total biomass, and overall plant height are observed as water levels continue to rise, indicating that the plants’ energy reserves or submersed tissues cannot sustain prolonged submergence [143,169,170].

##### Root Responses

Under periodic and continuous flooding stresses, plants develop distinct morphological and physiological plasticity in their root systems [43,164]. This developmental plasticity is more pronounced in young plants compared to mature ones [171]. Concurrently, aquatic macrophytes, such as *Typha domingensis*, *Phragmites australis* and *Eleocharis sphacelata*, exhibit an increase in aerenchyma gas spaces with rising water levels, enhancing their aeration potential as an adaptive response [172,173,174].

Simultaneously, plants can form adventitious or water roots [175] or produce more lenticels or stem hypertrophy [155,176] to gain more oxygen under flooding conditions. Studies have shown that root morphological adjustments, such as thickened roots, reduced lateral roots and shallow root systems, represent functional acclimations to flooding stress [177,178,179,180,181]. These architectural adjustments facilitate the radial oxygen loss from roots into the surrounding sediment, thereby oxygenating the rhizosphere of macrophytes [181,182]. In such root systems, root activity is maintained even under flooding pressure, as higher oxygen concentrations and oxidation-reduction potential (Eh) values typically occur in the upper soil layers [181,183]. Numerous studies have also documented that some aquatic macrophytes (such as *Phalaris* and *Spartina*) modulate biomass allocation among organs or increase their relative root length to root biomass ratio under waterlogged stress [118,184,185,186]. These adjustments collectively enhance nutrient uptake ability while minimizing the metabolic oxygen demand of the root system [118,184,185,186].

##### Leaf Responses

Flooding stress significantly influences leaf morphology, production rate, senescence dynamics, and biomass allocation [187,188,189]. Generally, plant leaves in deep-water environments exhibit larger size, greater mass, extended longevity, and reduced abundance compared to those in shallow-water conditions [186,187]. The leaf-size augmentation corresponds with an increased thickness of the leaf-surface-boundary layer, analogous to the thermal effects observed in sun-exposed leaves [190]. Edwards [149] indicate that several thick secondary walls and palisade layers can be found in emergent shoot tissues rather than in submerged shoots. With rising water levels, aquatic macrophytes produce elongated petioles and enlarged laminae leaves, although at a slower growth rate, resulting in fewer but longer-lived leaves per plant [191]. Concurrently, the petiolar mass per unit length of these aquatic macrophytes increases with water level [191]. Some floating-leaved plants adapt to temporary submergence by modulating petiole length to maintain leaf laminae above the water surface or in the air as water levels rise [187,188]. Similarly, emergent species demonstrate elongated petiole length several-fold greater under flooded conditions than in aerial environments [192].

While aquatic macrophyte leaf biomass exhibits a positive correlation with water depth, emergent leaves require greater construction costs than floating leaves [188]. However, emergent leaves demonstrate longer life spans than their floating counterparts [193]. Usually, older leaves typically exhibit higher mortality than younger leaves as the latter can more rapidly respond to a sudden water rise [160,194]. This leaf and shoot turnover strategy enables aquatic macrophytes to conserve energy for aerobic growth through the replacement of senescent tissues with new structures [153]. Furthermore, the withering of older leaves and shoots increases lacunae dimensions, thereby enhancing aeration potential [172,173]. However, shoots and leaves produced at high water levels—due to their thinner morphology—are more susceptible to mechanical failure or breakage [149,160,195].

##### Adventitious Root Responses of Woody Plants

Aquatic woody plants typically develop a suite of morphological adaptations to flooding stress, including thicker cuticles, multiple trunks, amphistomatic leaves, hypertrophied stems and lenticels, adventitious roots, large succulent water roots, and shallow root systems [171,196,197]. Among these responses, adventitious root development represents one of the most prominent and functionally significant morphological plasticity mechanisms because this adaptation is primarily regulated by the rootstock rather than the scion [198,199]. Supporting this, Wu et al. [6] suggest that following abrupt water-level increases in Lake Erhai, live willows (*Salix cavaleriei*) not only produce significantly more adventitious roots than dead individuals, but also initiate their highest adventitious roots at significantly higher positions along the stems. These aerenchymatous roots typically develop at stem nodes, hypocotyls, shoot bases and root apices [47]. While adventitious root production generally peaks in winter, its seasonal variation remains context dependent [200].

Adventitious root development is primarily determined by the hormones ethylene and auxin, with species-specific variation in its formation period [47]. Those roots play a crucial role in mitigating anoxia of aquatic woody plants by facilitating oxygen efficient transport from aerial tissues to submerged roots through their well-developed aerenchymatous network [201]. Additionally, adventitious roots can partially compensate for water and nutrient uptake functions of the original root systems because of their super hydraulic conductivity [55,202,203]. This functional redundancy effectively alleviates the physiological reliance on sediment-rooted systems, thereby enhancing plant resilience during prolonged inundation [170].

##### Biomass Allocation Responses

Biomass allocation represents a fundamental aspect of plant primary ecological strategy [204], and serves as a reliable proxy for competitive ability [205]. In response to water levels rising, aquatic macrophytes must optimize trade-offs among leaf expansion, root development, stolon growth and reproductive investment [118,206]. Consequently, numerous aquatic macrophytes prioritize aboveground biomass allocation, redirecting resources to vegetative structures to fuel accelerated vertical growth. This strategy is consistent with the elongation responses detailed previously [207,208,209]. Representative examples include *Juncus articulatus*, *Glyceria australis*, *Typha latifolia*, *Typha domingensis*, *Myriophyllum aquaticum*, *Eleocharis cellulosa*, *Spartina*, *Phalaris*, and so on.

Aquatic macrophytes derive greater benefits from this adaptive response because of enhanced gas exchange capability [210], a response mediated by a suite of mechanistic pathways [211]. However, increased shoot height in deep-water environments typically results in a compensatory reduction in shoot density [40]. Additionally, species with different life histories exhibit distinct biomass allocation patterns of their own in deep-water habitats [185]. For example, *J. articulatus* allocates a greater proportion of biomass to inflorescences compared to *G. australis* [185], while *T. latifolia* demonstrates higher leaf biomass allocation under flooding conditions. Meanwhile, *T. domingensis* maintains relatively stable leaf biomass proportions [212].

Furthermore, certain species maintain consistent biomass allocation patterns across plant organs with WLF [213,214]. While total plant biomass may remain unaffected by water depth in some aquatic macrophytes, the relative allocation ratios among leaves, roots and rhizomes exhibit strong correlations with water depth [186]. However, this morphological adaptation in biomass allocation represents a time-dependent process, which is influenced by multiple environmental and physiological factors [214]. For instance, *Eleocharis cellulosa* can demonstrates differential adjustment rates—modifying morphological indices within 3 weeks and achieving complete biomass reallocation after 9 weeks following water depth changes [149].

#### 3.2.4. Physiological Responses

##### Metabolic Responses

Aquatic macrophytes can adapt to flooding stress with a series of physiological processes, such as metabolic adjustments, photosynthetic modifications, respiratory reorganization and stress resistance mechanisms [47,143]. Upon submergence, whether partial or complete (applicable to emergent and rooted floating-leaved species), these plants employ multiple hypoxia avoidance strategies. These include enhanced root porosity [178], engagement of anaerobic respiration pathways [215], rapid shoot elongation, and the mobilization of carbohydrate reserves [216]. In deep-water conditions, aquatic macrophytes rely on anaerobic metabolism due to severe oxygen shortage [215]. Notably, a key differentiator between tolerant and sensitive species lies in their capacities to limit cytosolic acidosis and manage metabolic byproducts: species with low levels of submergence tolerance produce toxic ethanol as a byproduct of anaerobic respiration, while species with high levels of flooding tolerance exhibit a superior capacity to limit cytosolic acidosis [98,217,218].

Accordingly, under anaerobic metabolic conditions, flooding-stressed aquatic macrophytes accumulate numerous toxic substances, including ammonia, hydrogen sulfide, and, indirectly, toxic ethylene, which plants metabolize with difficulty [219]. These accumulated substances induce a significant decrease in leaf chlorophyll content, leaf water potential (specifically in emergent species), and soluble protein levels [220], ultimately triggering leaf senescence and premature abscission. This physiological deterioration results in marked reductions in photosynthetic efficiency and subsequent carbon fixation capacity [221,222], although ethylene and auxin can simultaneously stimulate adventitious root development [47]. Moreover, submergence-induced oxidative stress causes rapid over-accumulation of reactive oxygen species (ROS), leading to cellular senescence, programmed cell death and, ultimately, to plant damage or death [223].

##### Stress Resistance Mechanisms

Submergence stress generally induces a decline in the activities of antioxidative enzymes, such as superoxide dismutase (SOD), ascorbic acid oxidase (AAO), and malondialdehyde (MDA). Conversely, catalase (CAT) and peroxidase (POD) activities often increase in flooding-tolerant species [223]. This oxidative stress is frequently accompanied by proline accumulation, a well-established biomarker of plant stress and antioxidant defense [223]. However, species-specific responses do exist; in *Vallisneria spinulosa*, POD activity exhibits a positive correlation with flooding amplitude and frequency, while SOD activity remains stable [145]. Similarly, in *V. natans*, POD activity decreases under flooding condition, but MDA content increases when water transparency is enhanced by physical or chemical means, signifying an elevated risk of lipid peroxidation in plants [224]. Under abiotic conditions, proline typically accumulates because of mitochondrial dysfunction [225], yet this excess proline can serve as a protective resource or antioxidant reservoir for aquatic macrophytes upon stress relief [226]. Furthermore, some vitamin-based antioxidants, particularly ascorbic acid, vitamin E and other leaf antioxidant compounds, play important roles in mitigating flooding-induced damage by scavenging reactive oxygen species (ROS) [155]. These compounds demonstrate significant upgradation under flooding stress, thereby enhancing plant resilience [155].

##### Photosynthetic Responses

Light quantity and quality diminish with increasing water depth, and this is characterized by a declining ratio of red to green light [227,228,229]. Therefore, aquatic macrophytes (e.g., emergent species or littoral trees) decrease or cease their photosynthesis by closing their stomata under flooding stress [155,230], likely mediated by abscisic acid (ABA) accumulation [155,231]. This response occurs even in shallow water at high irradiance [229], although the light saturation points of some submerged macrophytes are very low [227,228,229,230,231,232]. Conversely, some emergent plants can maintain photosynthesis to some extend under water submergence [168], although this photosynthetic ability is very limited [233]. The soluble sugars used for anaerobic respiration in aquatic macrophytes result from transformation of reserved starch [233]. Therefore, deep water survival of aquatic macrophytes depends on total carbohydrate reserves, which serve as critical energy resources for plant anaerobic metabolism [234,235]. However, these reserves are ultimately depleted [169,236]. To delay this outcome, aquatic macrophytes can employ resource optimization strategies by maximizing their growth through biomass adjustment among organs to minimize resource consumption [118,237] or maintaining underwater photosynthesis [168,233]. However, both of these compensatory mechanisms decline progressively under prolonged flooding stress.

Additionally, aquatic macrophytes require substantial time to develop physiological or morphological responses to water flooding [12]. When flooding dynamics exceed the adaptive capacity of plant physiological and morphological adjustments, the resulting environment becomes detrimental to plant growth and development [209]. Furthermore, fast elongation constitutes a maladaptive strategy under certain flooding conditions as it depletes resource reserves while simultaneously increasing susceptibility to biotic stresses (diseases and insect pests) and structural vulnerabilities (mechanical damage from excessive elongated thin shoots) during post-flooding recovery [158,238].

##### Other Responses

Additionally, very few studies have investigated the mechanical resistance and stoichiometry responses of aquatic macrophytes to WLF. For instance, the mechanical resistance of aquatic macrophytes tends to decrease with increasing water depth [239]. Similarly, the stoichiometric characteristics of aquatic macrophytes are primarily influenced by species-specific traits rather than by variations in water depth or light availability [228].

#### 3.2.5. Reproductive Responses

##### Sexual Reproduction and Vegetative Propagation

Previous studies have shown that submergence significantly inhibits vegetative propagation, generative reproduction and even fruit maturation in aquatic macrophytes [136,240,241], reflecting deep-water stress. Congruently, flooding adversely affects seed survivorship, seed viability and subsequent seedling recruitment [98,242,243,244]. Accordingly, water drawdown is a prerequisite for successful germination and survival of various macrophytes, such as *Phalaris arundinacea* and *Iris pseudacor* [46], *Chara australis* [245] and some nymphaeid macrophytes [123]. In extreme cases, flooding can completely prevent reproductive structures from forming; for example, *Salvinia auriculata* fails to produce sporocarps on its ramets when flooded [246]. However, in some species such as *Ottelia alismoides*, neither the number of seeds per fruit nor the seed-setting rate is significantly affected along a water-depth gradient [247].

Along with fast elongation, aquatic macrophytes often adjust their reproductive and propagative strategies under flooding stress. These adjustments include delayed flowering, reduced vegetative propagation and increased flower production [151,241]. Such phenotypic plasticity in propagation strategy is closely linked to plant survival, growth, development, reproduction and even long-term population dynamics and succession [47]. The decrease in vegetative propagation is caused by inhibited tiller production, which results from limited availability of light, oxygen and nutrients under submergence. Under these conditions, limited energy reserves are preferentially allocated to support basic vegetative growth [136]. Conversely, in deep water, aquatic macrophytes exhibit a decline in tiller numbers but an increase in both flower numbers and the length of flowering shoots, suggesting a strategic preference for sexual reproduction under prolonged submergence [248,249,250]. As a result, higher seed production is expected, potentially leading to an increased number of new individuals in subsequent growing seasons [151]. However, some species can compensate for the reduction in sexual reproduction by enhancing vegetative propagation under submergence [151]. For example, *Vallisneria spinulosa* has been observed to lower its sexual reproduction investment while maintaining stable levels of asexual propagation in deep water environments [166].

With water depth increase, females of *V. natans* invest much more resources in reproduction than males, leading to an increase in sexual dimorphism. Then, the mortality in females is higher than in males due to increased reproductive expenditure at greater water depths, resulting in a male-biased sex ratio [251]. However, sex ratios in *V. natans* and *V. spinulosa* populations conformed to 1:1, whereas *V. denseserrulata* exhibits a pronounced female bias, potentially reflecting higher clonal ramet output by females [251]. These findings highlight the plasticity of both sexual dimorphism and sex ratios in dioecious plants, indicating that sex ratios are species-specific and shaped by sex-specific life-history strategies in response to local environmental conditions [251].

##### Seed Germination

It is generally considered that submerged species can germinate under water, whereas emergent species mainly germinate in moist terrestrial or shallow-soil conditions [252,253,254]. Previous research has shown that the germination percentages of two *Potamogeton* species do not differ significantly across flooded and moist environments [255]. In contrast, species such as *Hottonia palustris* and *Hottonia inflata* exhibit higher germination rates in waterlogged habitats compared to submerged environments [256,257]. However, emergent aquatic macrophytes can grow in deeper water habitats, primarily through vegetative propagation or by retaining the ability to germinate under water [258,259,260]. In contrast, flooding can promote the seed dispersal of aquatic macrophytes, a process that is predominantly mediated by hydrochory [46].

##### Trade-Off Between Growth and Reproduction

In summary, aquatic macrophytes must trade-off between somatic growth and reproductive output because of limited resources, a balance that is critically modulated by water depth [160,194]. For example, the spring shoot emergence of aquatic macrophytes is significantly inhibited when stored carbohydrates in belowground organs are greatly consumed under high flooding amplitude [212]. However, some scientists suggest that flooding-tolerant macrophytes allocate more energy to seed production [185], while drought-tolerant macrophytes typically allocate more resources to root development [261]. These interspecific variations in growth and propagation strategies are intrinsically linked to differences in their ecological traits, life-forms, and life histories [1,137,138,139]. However, our understanding of the optimal reproductive strategies employed under flooding stress remains conspicuously limited, highlighting the need for further comprehensive investigation [262].

In conclusion, the diverse survival strategies, morphological traits, physiological responses and propagation adaptations of aquatic macrophytes to WLF may reflect their species-specific evolutionary adaptations developed over a long evolutionary history [262].

## 4. Model Diagram of Aquatic Macrophytes Response to Flooding

Building upon the preceding synthesis, we constructed an integrative model diagram to explain a whole series of responses of aquatic macrophytes to flooding (Figure 3). Under negligible flooding regimes, aquatic macrophytes may exhibit minimal or no response, either because flooding levels do not negatively impact their physiology or because the disturbance falls below their minimum response threshold. As flooding intensity increases, aquatic macrophytes may adopt an escape strategy by fast elongation to maintain access to the water surface. However, this strategy is always accompanied by a decline in overall plant fitness. If flooding continues to intensify and exceeds the capacity of the escape strategy, aquatic macrophytes may instead resort to quiescence strategy, whereby growth and metabolic activity are suppressed to enhance survival under stress. This shift is typically associated with a sharp decrease in plant fitness. In some cases, aquatic macrophytes can selectively employ either the escape strategy or quiescence strategy, depending on the severity of flooding, thereby optimizing their resource allocation and enhancing survival. For plants unable to form a floating mat (floating mat formation strategy), especially emergent species, mortality is very likely to occur if they are unable to utilize escape or quiescence strategies effectively under extreme flooding stress. In such scenarios, conventional survival strategies become nonfunctional. However, the floating mat formation strategy enables aquatic macrophytes to escape from the direct stress of flooding stress and is associated with a significant improvement in plant fitness, regardless of the flooding intensity.

Similarly, aquatic macrophytes may show little or no morphological, physiological or propagative responses to low-amplitude flooding (Figure 3). As flooding intensity increases, aquatic macrophytes may initially recruit physiological responses to cope with the stress, often at the cost of a decline in plant overall fitness. This is because physiological processes are generally the most sensitive to environmental fluctuations, while morphological and reproductive alternations take much more time [12,166,214]. When flooding continues to increase, aquatic macrophytes may simultaneously employ morphological, physiological and reproductive responses to enhance tolerance. However, these compensatory mechanisms are invariably associated with a noticeable decrease in plant net fitness. Under conditions of extreme flooding pressure, however, aquatic macrophytes will die as their adaptive capacities become insufficient to sustain survival. Accordingly, aquatic macrophytes can maintain varying degrees of growth and development under low to moderate flooding stress, depending on species-specific traits and the combined use of the four response types (survival, morphological, physiological, reproductive, and, potentially, others). Nevertheless, the functions of these responses are very limited [20,56], particularly under extreme flooding conditions.

## 5. Conclusions

**Figure 4:**
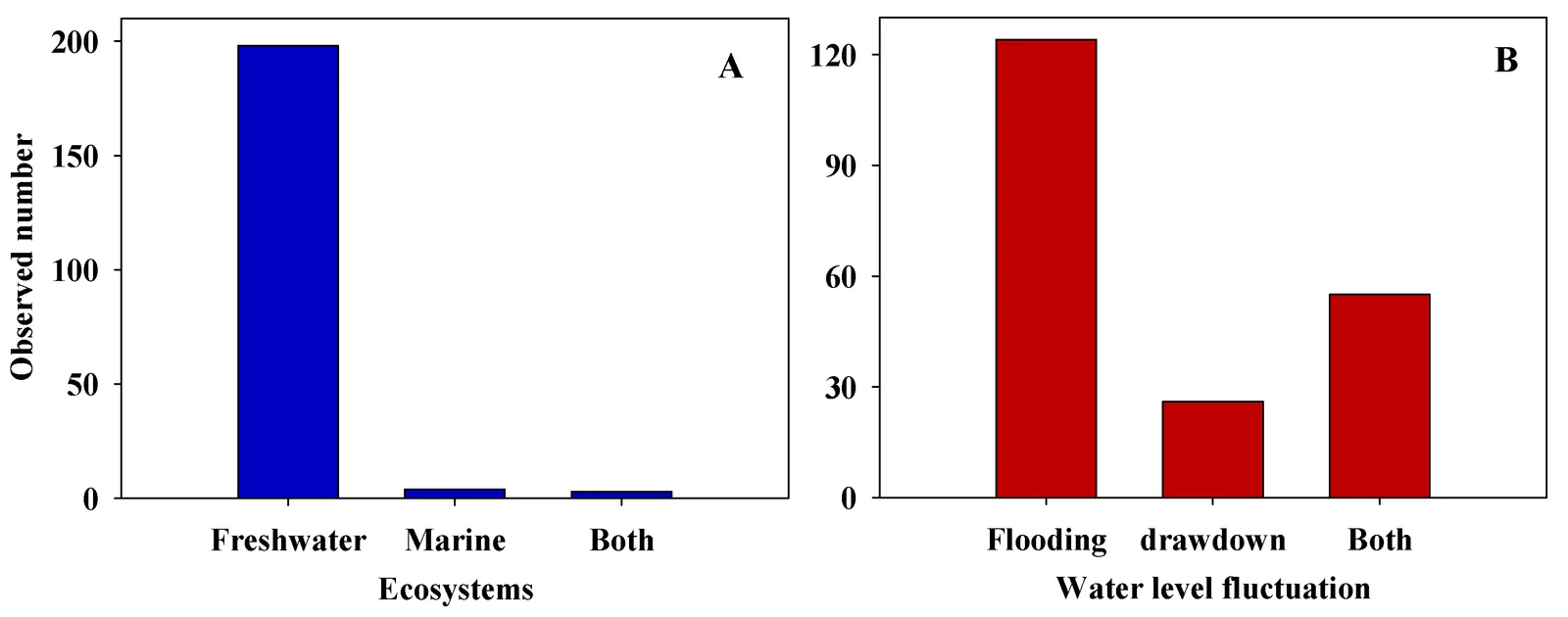
The observed number of collected studies in various ecosystems (**A**) and in different water-level-fluctuation types (**B**).

In this review, we synthesized the WLF effects on the environment where aquatic macrophytes establish, as well as their four primary adaptive strategies: survival, morphological, physiological and propagative responses. Based on our literature synthesis (see the Supplementary File), previous studies have predominantly focused on freshwater macrophytes and flooding-induced WLF, while drawdown-induced WLF and responses of marine macrophytes have received comparatively little attention (Figure 4). This disparity likely stems from the logistical complexities and extreme fluctuation amplitudes inherent in marine ecosystems, which pose significant challenges for experimental manipulation [262]. Moreover, the interplay between WLF and steep salinity gradients in coastal zones complicates the isolation of specific stress responses. Furthermore, the responses of invasive aquatic macrophytes to WLF (across all for strategy types) have been extremely underexplored [148]. However, these topics have attracted growing research interest and are likely to become a focal point in studies on macrophyte responses to WLF in the near future [263,264,265].

Accordingly, future research efforts should prioritize investigating the effects of drawdown WLF, responses in marine macrophytes, and the adaptive strategies of invasive aquatic macrophytes to WLF. Although environmental conditions, such as sediment stability, light availability, water pressure, pollutant exposure, wind and wave action, and fluctuations in oxygen and nutrient levels, become highly unfriendly to aquatic macrophytes when WLF occurs, aquatic macrophytes can adapt through rapid adjustments in their survival, morphological, physiological and propagative responses. While the adaptive capacity of aquatic macrophytes to flooding stress is generally limited, and life-form- or species-dependent, it varies with the intensity and duration of flooding. This limitation can lead to dominant species turnover or community succession, even over relatively short timescales in aquatic ecosystems [20,134]. Because plant communities comprise species with divergent adaptive traits, ecological amplitudes, niches and life-history strategies, their net response to water-level fluctuations is often non-linear and species-specific. Consequently, shifts in hydrological regimes can lead to rapid alterations in community composition and succession, favoring taxa with broader tolerances [20,266]. Accordingly, while aquatic macrophytes may benefit from an appropriate WLF [267,268], they are highly vulnerable to strong and prolonged WLF events. Accordingly, careful and precise water-level regulation can be employed to maximize the good ecological functions and services provided by aquatic ecosystems.

## 6. Future Research Need

Based on our review of the literature, we have identified six key knowledge gaps that warrant further investigation to improve our understanding of how aquatic macrophytes respond to water-level fluctuations (WLF).

(1) Do invasive aquatic species exhibit more effective adaptive responses to WLF than native aquatic macrophytes, thereby increasing the susceptibility of aquatic ecosystems to plant invasions [20]?
(2) What species-specific traits enable marine macrophytes to adapt to extreme WLF events in marine ecosystems, compared to their freshwater counterparts?
(3) How do aquatic macrophytes cope with and survive heightened WLF disturbances driven by climate change [2,7,8,9]?
(4) While we synthesized the responses of aquatic macrophytes to individual WLF factors, how do they respond to combined stressors, including WLF and co-occurring pressures such as pollution and toxicity?
(5) What is the optimal regulated water depth for maximizing ecological functions and services of a given species or community in aquatic ecosystems?
(6) Through which adaptive molecular and genetic mechanisms do aquatic macrophytes respond to water-level fluctuations (WLF)?

## Data Availability

Please contact the author for materials requests, and the materials will be available online upon your request.

## Supplementary Materials

The following supporting information can be downloaded at: https://www.mdpi.com/article/10.3390/plants15172673/s1, Table S1: The 205 studied article in this review.
